# Hyaluronic Acid-Functionalized Gadolinium Oxide Nanoparticles for Magnetic Resonance Imaging-Guided Radiotherapy of Tumors

**DOI:** 10.1186/s11671-020-03318-9

**Published:** 2020-04-25

**Authors:** Chaoyang Wu, Rong Cai, Tian Zhao, Lin Wu, Lirong Zhang, Jie Jin, Lixia Xu, Pengfei Li, Tao Li, Miaomiao Zhang, Fengyi Du

**Affiliations:** 1 Department of Oncology, The Affiliated People’s Hospital, Jiangsu University, Zhenjiang, 212002 People’s Republic of China; 2 School of medicine, Jiangsu University, Zhenjiang, 212013 Jiangsu People’s Republic of China; 3 Department of Radiology, Affiliated Hospital of Jiangsu University, Zhenjiang, 212013 People’s Republic of China

**Keywords:** Hyaluronic acid, Gadolinium, Radiosensitization, MR imaging

## Abstract

Inaccuracy localization and intrinsic radioresistance of solid tumors seriously hindered the clinical implementation of radiotherapy. In this study, we fabricated hyaluronic acid-functionalized gadolinium oxide nanoparticles (HA-Gd_2_O_3_ NPs) via one-pot hydrothermal process for effective magnetic resonance (MR) imaging and radiosensitization of tumors. By virtue of HA functionalization, the as-prepared HA-Gd_2_O_3_ NPs with a diameter of 105 nm showed favorable dispersibility in water, low cytotoxicity, and excellent biocompatibility and readily entered into the cytoplasm of cancer cells by HA receptor-mediated endocytosis. Importantly, HA-Gd_2_O_3_ NPs exhibited high longitudinal relaxivity (*r*_1_) 6.0 mM^−1^S^−1^ as MRI contrast agents and radiosensitization enhancement in a dose-dependent manner. These finds demonstrated that as-synthesized HA-Gd_2_O_3_ NPs as bifunctional theranostic agents have great potential in tumors diagnosis and radiotherapy.

## Introduction

Radiotherapy has been extensively applied in cancer, which involves high-energy X-ray and deposition of irradiation doses at tumor sites by causing free radical damage or DNA damage [1–4]. However, poor radiosensitivity, inaccuracy of tumor localization and poor discrimination between lesions, and normal tissues causing irradiation side effects limit the clinical implementation of radiotherapy [5]. Therefore, it is important to develop methods for enhancing tumor radiosensitivity while minimizing systemic side effects. A combination of nanotechnology and radiotherapy is a well-established priority for radiosensitization.

In recent years, nanotechnology has been considered an attractive strategy for cancer diagnosis and therapy [6–11]. One of the principal functions of nanoparticles (NPs) is an accurate tumor targeting based on the selective accumulation of NPs in tumor tissues via passive targeting, the enhanced permeability and retention (EPR) effect, active targeting, and the prolonged circulation time [12–14]. Our previous work demonstrated that doping carbon NPs with heteroatoms effectively tunes their intrinsic properties and introduces beneficial features [15–17]. Interestingly, NPs can be used as radiosensitizers [18]. Heavy metal (with high Z-elements) NPs (e.g., Au, Bi, Gd) as promising radiosensitizers can be used in radiosensitizing therapy because of their high X-ray photon capture cross-section and Compton scattering effect [19]. When X-rays interact with high Z nanoparticles, Auger electrons, and photoelectrons, the release of secondary electrons injure cancer cells, providing dose enhancement during radiation therapy [20]. To date, gadolinium-based NPs (GdNPs) are shown to be effective MRI contrast agents (CAs) [21, 22] and differentiate normal tissue from diseased tissue and lesions in a noninvasive manner and in real-time. These agents shorten longitudinal relaxation time to affect longitudinal relaxivity *r*_*1*_ [23], and their inaccurate targeting frequently results in side effect. It is well known that hyaluronic acid (HA) is a major ligand and a drug delivery carrier to target sites with HA receptors such as cluster determinant 44 (CD44) [24–26].

Based on the results of previous studies, we used HA as a targeting ligand to functionalize Gd_2_O_3_ NPs with dual functions: effective tumor-targeting MRI CAs and radiosensitizers to overcome inherent radioresistance and the inaccuracy of tumor localization. In addition, HA-Gd_2_O_3_ NPs display high longitudinal relaxivity (*r*_*1*_) as promising MR imaging agents with better MR imaging quality. Compared with currently available CAs [27, 28], the as-resultant HA-Gd_2_O_3_ NPs show three significant advantages: firstly, the HA-Gd_2_O_3_ NPs exhibit favorable biocompatibility due to using the natural extracellular matrix as the precursors. Secondly, HA functionalization significantly improves tumor targeting and reduces the side effects. Finally, the HA-Gd_2_O_3_ NPs possess bifunctional capability in diagnosis and therapy. In order to verify the effectiveness and explore the mechanism of radiosensitization enhancements, we evaluated the radiosensitizing effect of HA-Gd_2_O_3_ NPs on tumor cell viability, the cell cycle, and apoptosis.

## Results and Discussion

### Preparation and Characterization of HA-Gd_2_O_3_ NPs

**Scheme 1:**
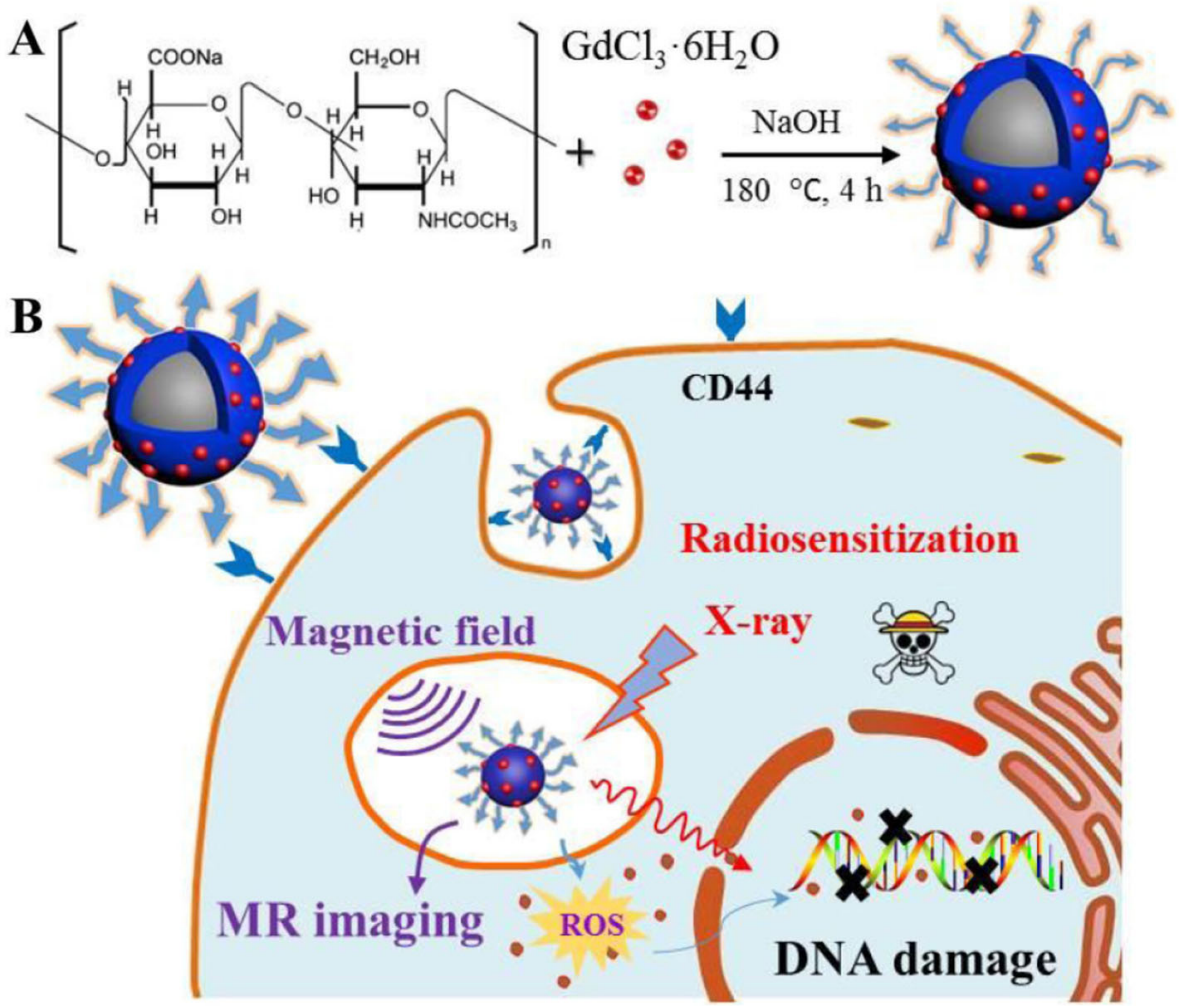
Schematic synthesis of HA-Gd_2_O_3_ NPs from the hydrothermal method (A) and the following biomedical applications (B)

**Fig. 1:**
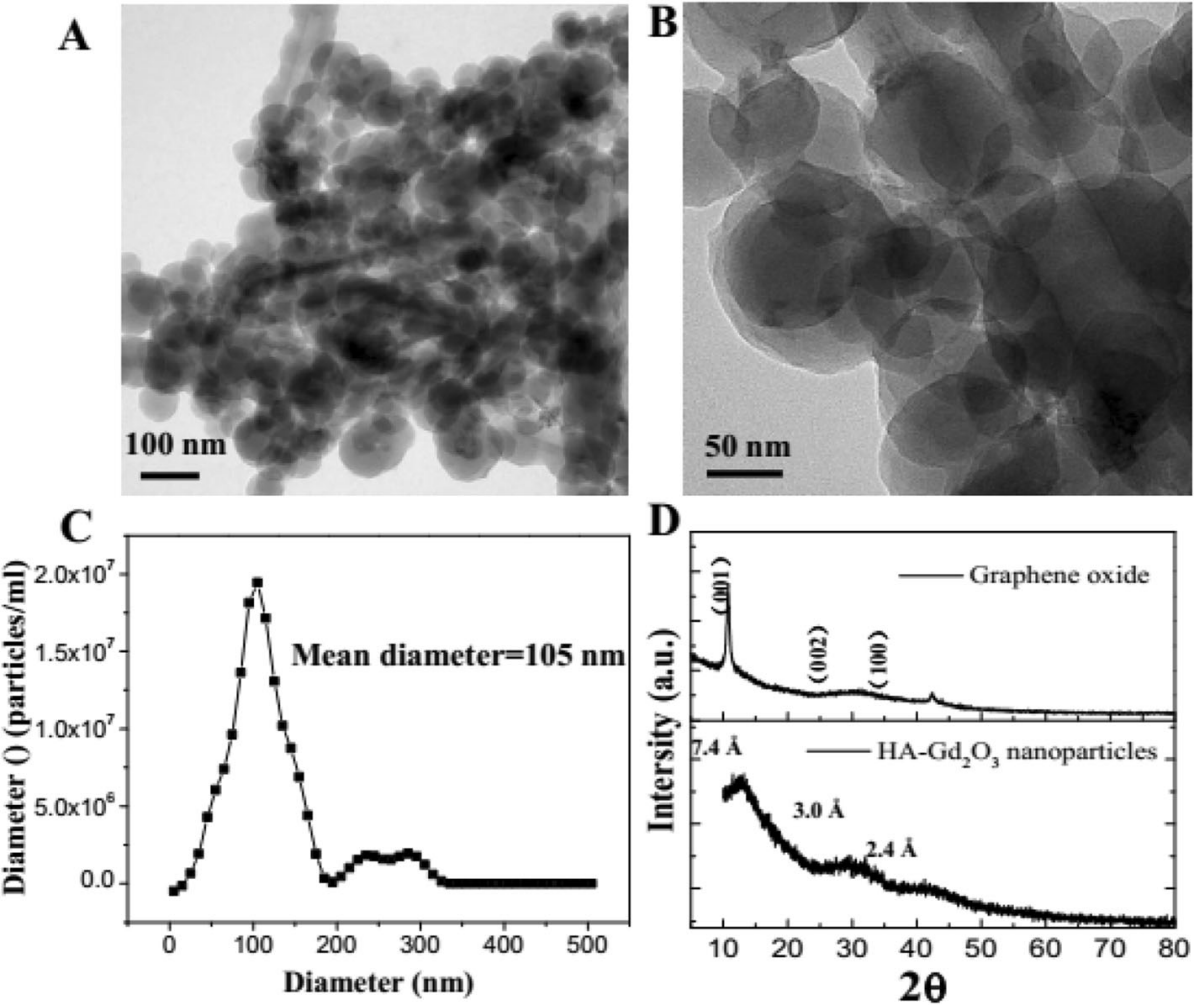
Characterization of HA-Gd_2_O_3_ NPs. Low (**a**) and high (**b**) magnification TEM images. The diameter distribution (**c**) and XRD patterns (**d**) of HA-Gd_2_O_3_ NPs

In this study, HA-Gd_2_O_3_ NPs were successfully prepared using a simple hydrothermal process as shown in Scheme 1. Particle sizes were analyzed by dynamic light scattering, while the morphological examination was conducted by transmission electron microscopy. As shown in Fig. 1a, HA-Gd_2_O_3_ NPs exhibited uniform dispersion and discrete quasi-spherical shapes without apparent aggregation. The average diameters of the HA-Gd_2_O_3_ NPs were 105 nm. Compared to normal tissues, the capillary endothelial permeability of tumor tissues was increased and the endothelial gap was 100–600 nm [29]. Therefore, NPs with desirable sizes were easily immersed in tumor tissues, and they may dramatically increase the efficiency of passive targeting drug delivery.

The phase structure of HA-Gd_2_O_3_ NPs was investigated by XRD using graphite oxide (GO) power as a control. As shown in Fig. 1d, one main diffraction peak (2*θ* = 12.04°) and two minor peaks (2*θ* = 29.6°, 42.1°) were observed in the HA-Gd_2_O_3_ NP diffraction pattern, which corresponded to the characteristic peaks of GO (100 plane) and graphite (002 plane), respectively. The main lattice spacing of HA-Gd_2_O_3_ NPs showed smaller spacing (0.73 nm calculated by the Bragg formula) than the GO spacing of *d*_001_ = 0.85 nm, where the upward shift in the peak position may be attributed to the decrease in spacing between the sp^3^ layers. All broad diffraction peaks demonstrated that HA-Gd_2_O_3_ NPs had an amorphous structure, which may be attributed to the highly disordered carbon and decrease in sp^2^ (C–C) layer spacing during the carbonization process. The result further indicated HA-Gd_2_O_3_ NPs with a poor crystalline nature possessed a heterogeneous multi-layered structure, which was consistent with our previous reports regarding carbon dots [30, 31].

### Chemical Structure and Surface Composition of HA-Gd_2_O_3_ NPs

**Fig. 2:**
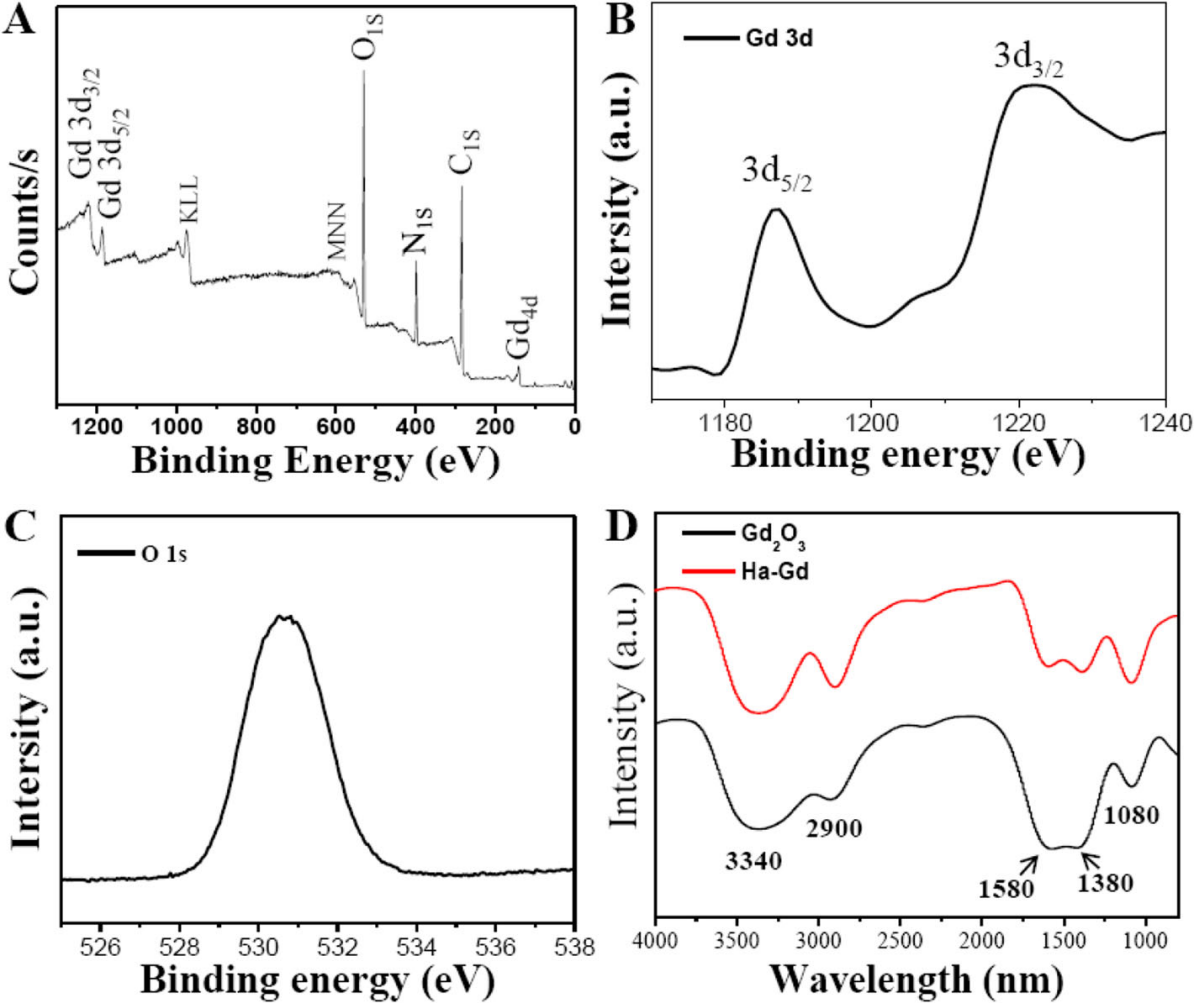
The chemical structure and composition of the HA-Gd_2_O_3_ NPs. **a** The full-scan XPS spectrum of HA-Gd_2_O_3_ NPs. **b** Gd_3d_ spectrum. **c** O_1S_ spectrum. **d** The FTIR spectrum of HA-Gd_2_O_3_ NPs

Surface functional groups and composition of HA-Gd_2_O_3_ NPs were investigated using Fourier transform infrared spectrum and X-ray photoelectron spectroscopy. As shown in Fig. 2a, the XPS spectrum showed four typical peaks at 284.0, 400.0, 530.6, and 1188.5 eV, indicating that HA-Gd_2_O_3_ NPs were mainly composed of gadolinium, carbon, oxygen, and hydrogen elements. The high-resolution spectrum of Gd_3*d*_ (Fig. 2b) revealed the presence of two strong peaks at 1187.5 eV and 1221 eV, corresponding to a spin-orbit splitting of 32 eV, corresponding respectively to the 3*d*_5/2_ and 3*d*_3/2_ energy levels of Gd. These observations were in good agreement with previous reports of HA-Gd_2_O_3_ NPs. The O (1*s*) spectrum shown in Fig. 2c was dominated by one major peak positioned at 531.4 eV, which corresponded to the bond between O^2−^ and Gd^3+^. FITR spectroscopy was conducted for both naked and HA-Gd_2_O_3_ NP samples (Fig. 2d). For the naked samples, as shown in Fig. 2a, the characteristic absorption bands of *v*_as_ O–C–O at 1580 and 1380 cm^−1^ revealed the presence of a carbonate group. The broad peaks at 3340 and 2900 cm^−1^ were attributed to the O–H and C–H stretching vibrations, respectively, which corresponded to the surface-adsorbed water. For HA-Gd_2_O_3_ NPs, the stretching vibrations of COO^−^ at 1580 and 1380 cm^−1^ were enhanced, indicating the introduction of the carboxylic group of hyaluronic acid. These results revealed that the functional groups of HA-Gd_2_O_3_ NPs mainly contained certain numerous carbonyl, carboxylate, and hydroxyl groups. The presence of these functional groups located on the surface endowed HA-Gd_2_O_3_ NPs with excellent dispersibility in water. Importantly, the Gd ion embedded in the surface of the NPs may be useful for MR imaging and radiosensitization, which dramatically prevented the Gd ion from leaking into the surrounding environments.

### Biocompatibility of HA-Gd_2_O_3_ NPs

**Fig. 3:**
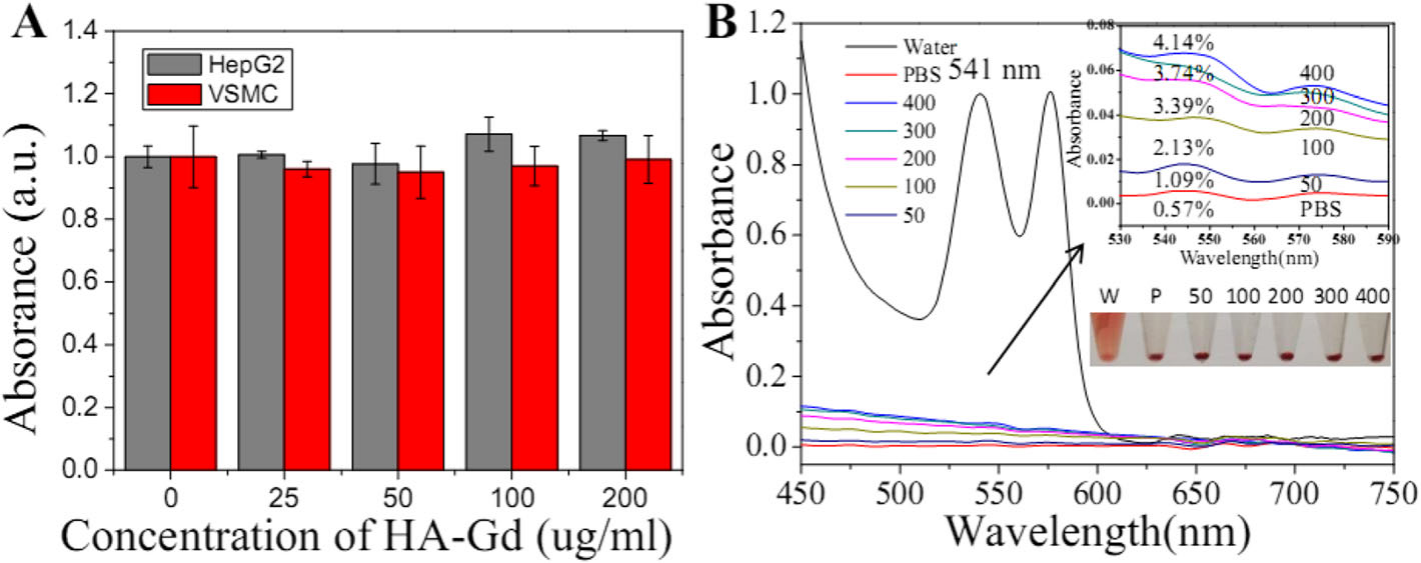
**a** Effect of HA-Gd_2_O_3_ NPs on the viability of HepG2 and VSMC cells using the CCK-8 assay. **b** Hemolytical activity of the HA-Gd_2_O_3_ NPs at different concentrations (50, 100, 200, 300, and 400 μg/mL). PBS and water were used as the negative and positive control, respectively

Biocompatibility of HA-Gd_2_O_3_ NPs, as potential biomedical agents, is critical for their biomedical application. First, the inherent cytotoxicity of HA-Gd_2_O_3_ NPs was assessed in HepG2 and VSMC cells using the CCK-8 assay. As shown in Fig. 3a and S. Fig. 1, the HA-Gd_2_O_3_ NPs did not exhibit obvious cytotoxicity up to 3 days. Even at a concentration of 200 μg/mL after a 24 h exposure time, cell viability was approximately 90%. Second, the hemocompatibility of HA-Gd_2_O_3_ NPs in vivo was estimated using a hemolysis assay. As shown in Fig. 3b, we observed hemolysis of red blood cells in the water (positive control). In contrast, no obvious hemolysis was observed after HA-Gd_2_O_3_ NP incubation at different concentrations from 0 to 200 μg/mL for 2 h, which was similar to the results for PBS solution (negative control). Compared to the negative control, the percentage of hemolysis at different concentrations of HA-Gd_2_O_3_ NPs was slightly evaluated based on the absorbance of the supernatant at 541 nm. The results showed that the hemolysis percentages of HA-Gd_2_O_3_ NPs were all less than 3% in the studied concentration, verifying their favorable hemocompatibility. In order to examine the potential toxicity, HA-Gd_2_O_3_ NPs were intravenously injected into Balb/c mice (as seen in S. Fig. 2). After 1 week, these mice were executed, and the bladder, kidney, and spleen were harvested for pathological section analysis. As shown in S. Fig. 2, the result of pathological sections showed that there was no observably lesion or inflammatory response in the organs of HA-Gd_2_O_3_ NP-treated mice. These results clearly indicated that the as-synthesized HA-Gd_2_O_3_ NPs had favorable cytocompatibility and good hemocompatibility.

### MRI Phantom Study

**Fig. 4:**
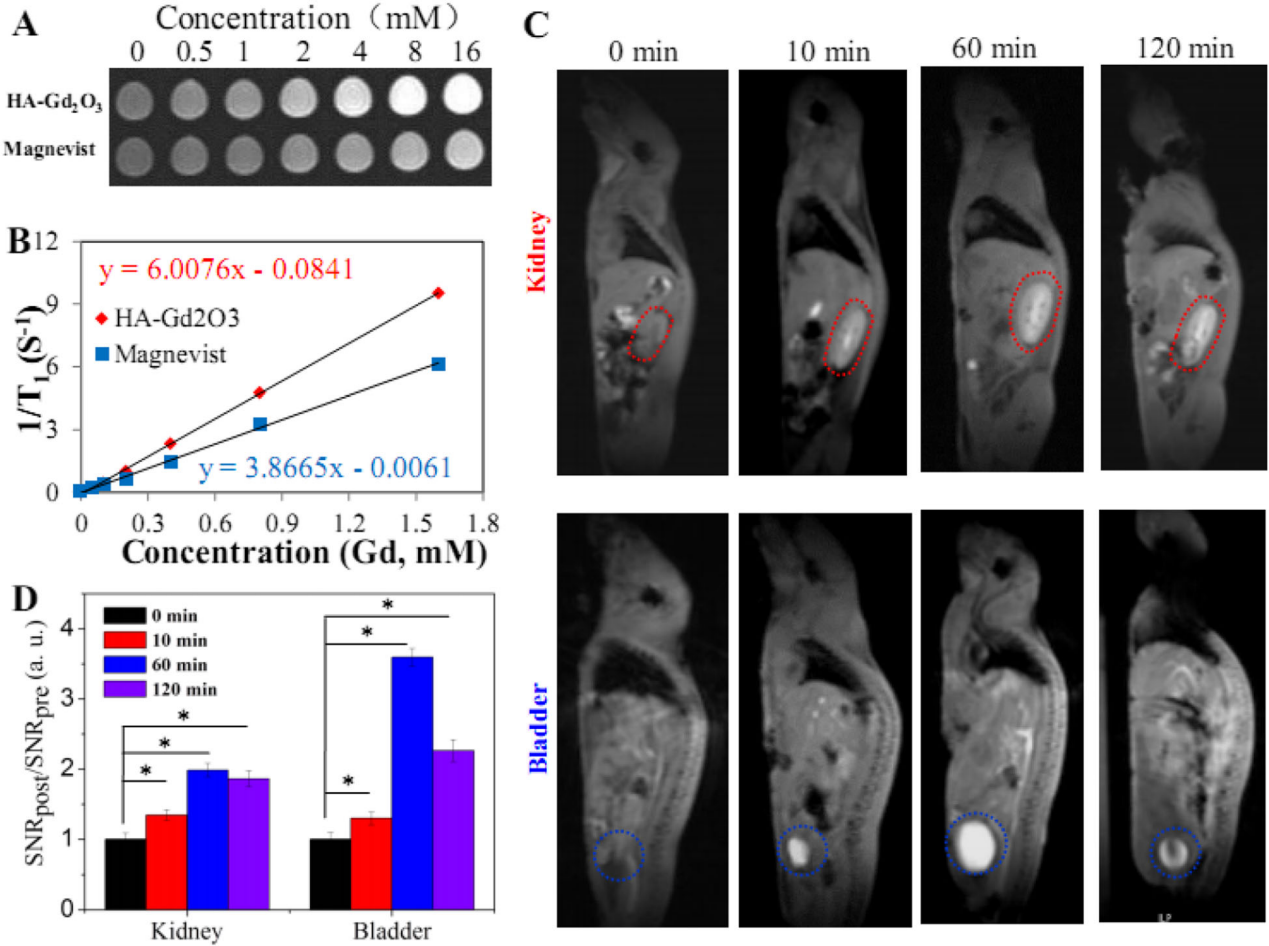
**a** T_*1*_ phantom images of the HA-Gd_2_O_3_ NPs with different concentrations of total Gd ions. **b** The corresponding plots of 1/T_*1*_ against concentrations of total Gd ions. **c** In vivo T_*1*_ MR imaging and analysis of mice after intravenous injection of HA-Gd_2_O_3_ NPs as contrast agents. **d** Quantification of signal changes (SNR ratio) in the bladder and kidney at different time points after administration (*n* = 3). *p < 0.05

The longitudinal (*T*_1_) relaxation times of HA-Gd_2_O_3_ NPs were investigated in vitro together with the commercial contrast Magnevist (Gd-DTPA) as a control using phosphate-buffered saline (pH = 7.4, 0.2 M). With increasing Gd concentrations, the signal intensity of *T*_1_-weight phantom images clearly increased, indicating that all samples could generate MRI contrast enhancement on *T*_1_-weighted sequences (Fig. 4a). Furthermore, plots of the signal intensity versus inversion time gave *T*_1_-relaxation times of each contrast agent at specific concentrations. As shown in Fig. 4b, the *r*_1_ value of HA-Gd_2_O_3_ NPs was measured to be 6.0 mM^−1^S^−1^, which was significantly higher than that of Magnevist (3.86 mM^−1^S^−1^) under the same conditions. The enhanced *r*_1_ may be ascribed to the much higher hydrodynamic radius and surface area of HA-Gd_2_O_3_ NPs; therefore, more Gd atoms doped in the lattice of NPs became accessible to water molecules, shortening the longitudinal relaxation and enhancing the *r*_*1*_ value.

### MR Imaging In Vivo

In order to determine the potential applications in vivo, the MR imaging performance and circulation fate of HA-Gd_2_O_3_ NPs (10 mg/kg) were investigated using an MRI scanner with normal BALB/c mice as a model. Compared with pre-injection images (Fig. 4c), the brighter regions of the kidney and bladder as indicated by the dashed circles were clearly observed at 10 min post-injection, demonstrating that HA-Gd_2_O_3_ NPs could be used as CAs enhance *T*_1_ relaxation in these major organs in vivo. Importantly, the contrast enhancement of HA-Gd_2_O_3_ NPs was maintained to 60 min post-injection, which was much longer than that of Gd complex small molecules (half-life of about several minutes in small animals) [27], indicating that HA-Gd_2_O_3_ NPs possessed longer retention times in vivo than the commercial contrast. To analyze the MR contrast effect quantitatively, we calculated the signal-to-noise ratio (SNR) by finely analyzing regions of interests of the MRI images and calculated the values of SNR_post_/SNR_pre_ to represent the relative signal enhancement (RSE) (Fig. 4d). The RSE values of HA-Gd_2_O_3_ NPs in the kidney were 1.35, 1.99, and 1.86. Similarly, the RSE values of HA-Gd_2_O_3_ NPs in the bladder were higher than those in the kidney (1.29, 3.59, and 2.26). HA-Gd_2_O_3_ NPs with big size (more than 100 nm) displayed long circulation times, then may be eliminated with biliary-intestinal pathway according to previous results [32]. Moreover, the prolonged circulation time in vivo may have increased passive targeting in tumors by enhancing the EPR effect.

### Tumor Imaging

**Fig. 5:**
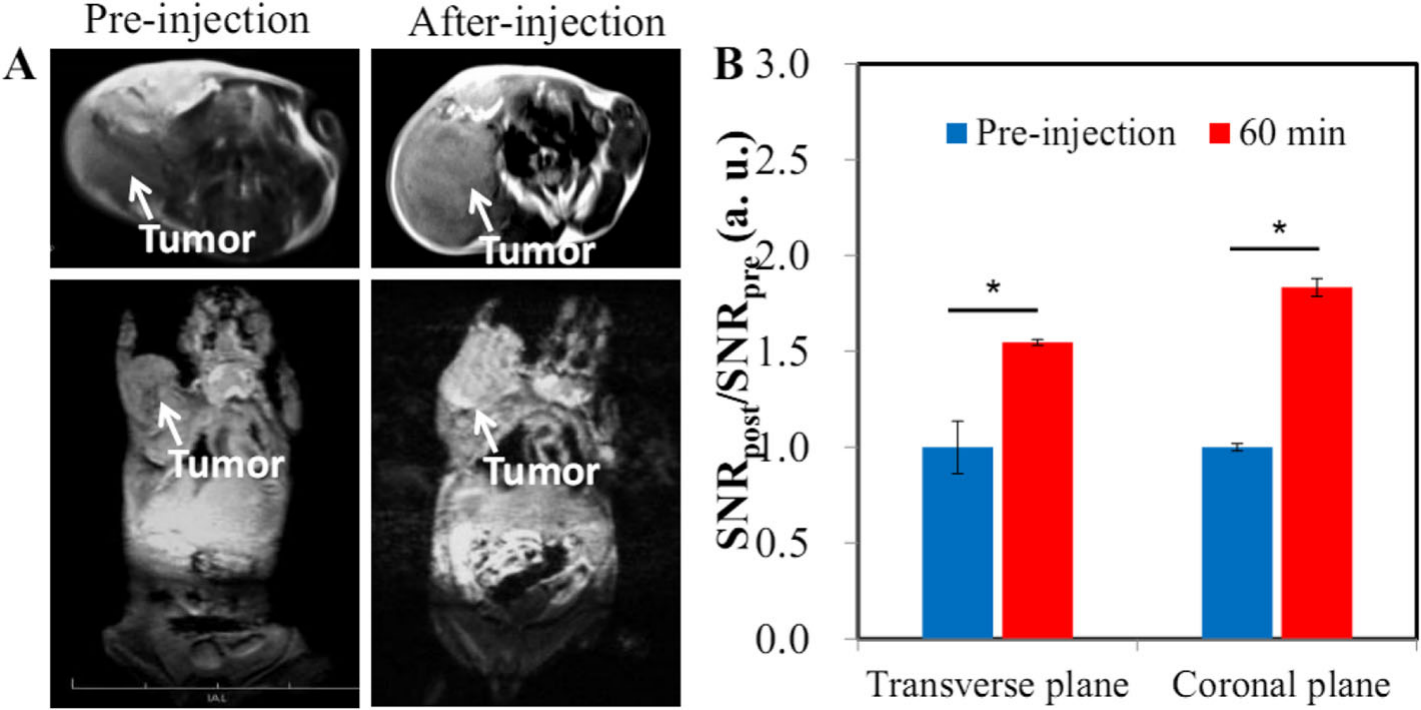
*T*_1_-weighted MR imaging (**a**) and corresponding quantificational analysis (**b**) of Heps mouse liver cancer xenograft tumors after intravenous injection of HA-Gd_2_O_3_ NPs

HA-Gd_2_O_3_ NPs with excellent MR imaging performance and long circulation time provide a great opportunity for imaging tumors through an EPR effect. We thus established subcutaneous liver tumor models to investigate whether hepatocellular carcinomas (HCCs) can be detected by HA-Gd_2_O_3_ NP-enhanced MRI. After intravenous injection of the HA-Gd_2_O_3_ NPs, we observed that the subcutaneous tumor region becomes brighter than the surrounding tissue as seen by both coronal and transverse scanning (Fig. 5a). The RES of the subcutaneous tumor dramatically reached 1.54 and 1.83 with transverse and coronal scanning, respectively, indicating the effective accumulation of the HA-Gd_2_O_3_ NPs in the tumor region through the EPR effect. These findings demonstrate that HA-Gd_2_O_3_ NPs are highly sensitive for MRI visualization of tumors. Many studies showed that the long circulation time could promote the efficiency of passive targeting through leaky vasculature of solid tumors [33].

### Radiosensitization Enhancement of HA-Gd_2_O_3_ NPs

**Fig. 6:**
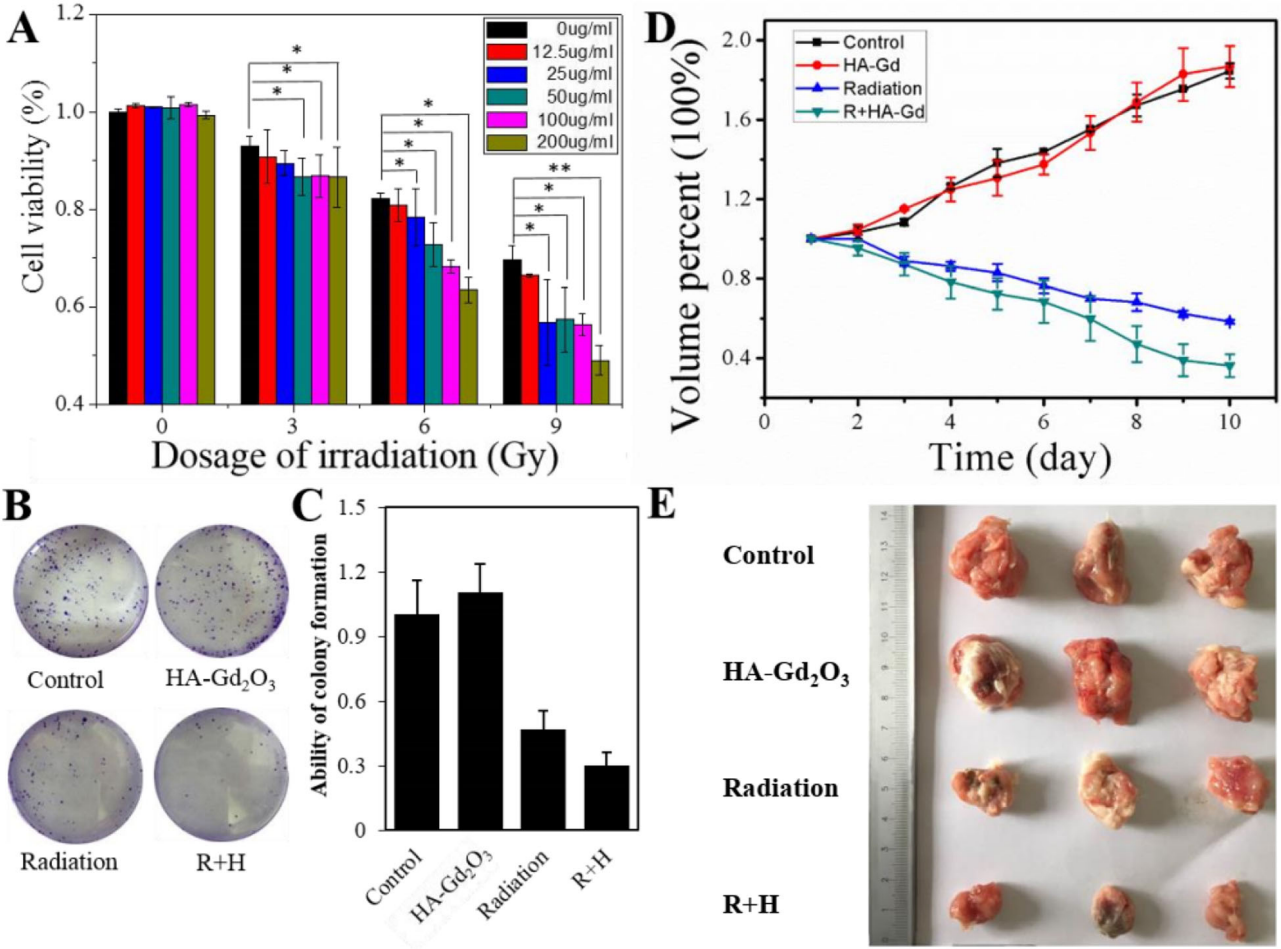
The radiosensitization effect of HA-Gd_2_O_3_ NPs in vitro. **a** The synergistic effect of HA-Gd_2_O_3_ NPs (0 μg/mL, 12.5 μg/mL, 25 μg/mL, 50 μg/mL, 100 μg/mL, and 200 μg/mL) and X-ray radiation (0 Gy, 3 Gy, 6 Gy, and 9 Gy) on the viability of HepG2 cells using the CCK-8 assay. *p < 0.05, **p < 0.01, the difference was statistically significant. **b** The colonies survival assays of HepG2 cells treated with HA-Gd_2_O_3_ NPs and X-ray radiation and **c** the corresponding quantitative analysis of clonegenesis abilities. **d** Tumor volume growth curves of different groups of mice after different treatments. Groups: **a** control, **b** radiation, **c** HA-Gd_2_O_3_ NPs, and **d** radiation + HA-Gd_2_O_3_ NPs. **e** Photos of tumors collected from different groups of mice at the end of treatment

The excellent performance of HA-Gd_2_O_3_ NPs as a *T*_1_ contrast agent prompted us to determine its location in the radiosensitization of tumors accurately. First, whether dose enhancement occurred with this combination treatment was explored using a CCK-8 assay. As depicted in Fig. 6a, single HA-Gd_2_O_3_ NPs (concentration 0–200 μg/mL) did not significantly influence the viability of HepG-2, but X-ray (range 0–9 Gy) irradiation decreased HepG-2 cell viability to less than 70% at 9 Gy. Importantly, a combination of X-ray irradiation with HA-Gd_2_O_3_ NPs dramatically decreased HepG-2 cell viability to less than 50%, particularly at a concentration of 200 μg/mL at 9 Gy. Next, a clonogenic assay was conducted to evaluate the cell viability in HepG-2 cells following a combination of X-ray irradiation and HA-Gd_2_O_3_ NPs. As shown in Fig. 6b, single HA-Gd_2_O_3_ NPs had no dramatic influence on HepG-2 cell colony formation, but X-ray irradiation decreased the HepG-2 cell colony formation to 46.7%. However, treatment with X-ray irradiation and HA-Gd_2_O_3_ NPs notably inhibited cell colony formation viability to 29.8%. The corresponding images further verified the radiosensitization of HA-Gd_2_O_3_ NPs against HepG-2 cells.

The effectiveness of radiotherapy in vitro encouraged us to apply HA-Gd_2_O_3_ NPs combined with the irradiation to control tumor growth in tumor-bearing mice. The volumes of tumors in the control and single HA-Gd_2_O_3_ NP treatments grew rapidly and both increased by 180%. Compared with the single irradiation treatments 58%, HA-Gd_2_O_3_ NPs combined with radiation showed efficient tumor inhibition by 38% after 10 days of irradiation (Fig. 6d). The tumor was photographed, and the average tumor volumes in each group showed that the average tumor volume in group (a) was the highest while that in group (d) was the lowest among all groups (Fig. 6e). These results reveal that the as-prepared HA-Gd_2_O_3_ NPs are promising for the inhibition of tumors growth under X-ray irradiation.

**Fig. 7:**
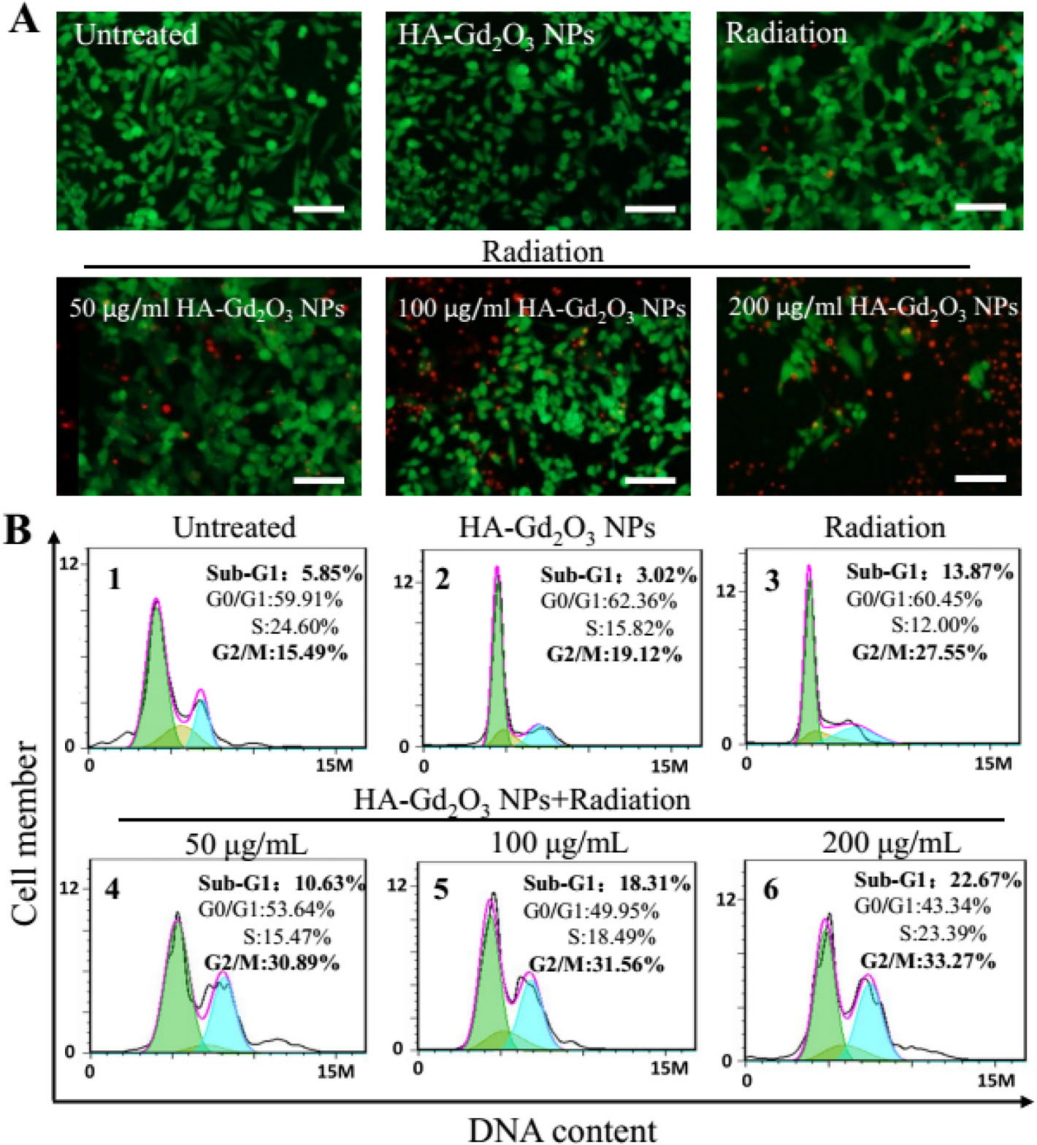
Radiation dose enhancement of HA-Gd_2_O_3_ NPs. **a** Live-dead staining of HepG2 cells. Green (fluorescein diacetate stain) = live cells, red (propidium iodide stain) = dead cells. Scale bars = 200 mm. **b** The cell cycle distribution after different treatments were analyzed by quantifying DNA content using flow cytometry assay

**Fig. 8:**
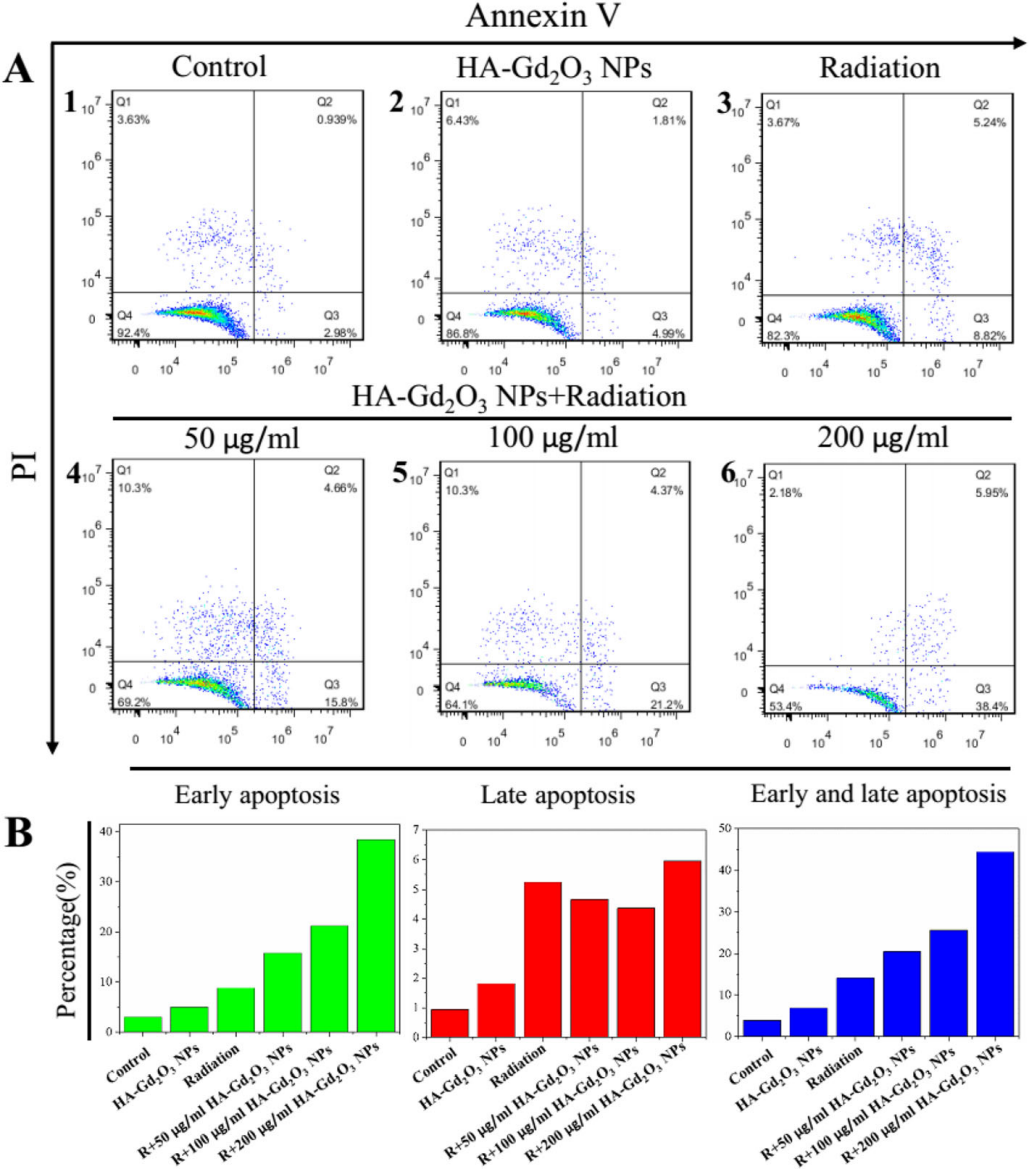
The apoptosis analyses of HA-Gd_2_O_3_ NPs. **a** The apoptosis levels of HepG-2 cells at 24 h after treatment of X-ray radiation. b The corresponding quantitative analysis

Flow cytometry assay and live/dead staining assay were conducted to further understand the radiosensitization mechanism in the combined treatment of HA-Gd_2_O_3_ NPs and X-ray radiation. As shown in Fig. 7a, intensive green fluorescence without red was observed in HepG-2 cells treated with single HA-Gd_2_O_3_ NPs, which verified high cell viability. A small amount of red fluorescence was observed after X-ray radiation treatments, indicating a low level of cell apoptosis. However, strong red fluorescence was observed following combination treatment of HA-Gd_2_O_3_ NPs (200 μg/mL) and radiation, demonstrating that HA-Gd_2_O_3_ NPs could dramatically enhance X-ray irradiation-induced cell apoptosis. The radiosensitization mechanism of HA-Gd_2_O_3_ NPs was further investigated using flow cytometry. As depicted in Fig. 7b, single radiation and HA-Gd_2_O_3_ NPs induced 27.55% and 19.12% of G2/M phase arrest, respectively. However, combination treatment of HA-Gd_2_O_3_ NPs and radiation notably increased G2/M phase arrest, ranging from 30.89 to 33.27% in a dose-dependent manner. Meanwhile, the combination treatment induced apoptotic cell death from 10.63 to 22.67%, as reflected by the sub-G1 proportions when the single HA-Gd_2_O_3_ NPs and radiation induced 3.02% and 13.87%. To further investigate the possible mechanism of cell death, an Annexin V-EGFP/PI method was conducted by the flow cytometry. Annexin V-EGFP emission signal was plotted on the *x*-axis, while PI emission signal was plotted on the *y*-axis (Fig. 8). The quantities of necrosis cells, early apoptosis cells, late apoptosis cells, and living cells were determined by the percentage of Annexin V^−^/PI^+^(Q3), Annexin V^+^/PI^+^ (Q2), and Annexin V^−^/PI^−^ (Q4), respectively. The apoptotic percentage of the cells in the control group and single HA-Gd_2_O_3_ NP (100 μg/mL) group were less than 5%, resulting in subtle influence. Compared with single-radiation treatment groups (8.8%), the apoptosis rate of the combination of HA-Gd_2_O_3_ NPs and radiation increased with the concentration increased ranging from 50 to 200 μg/mL. Especially, the early apoptosis rate of cells obviously increased to 33.2%, and the early and late apoptosis reached 44.3% when the concentration is 200 μg/mL. Generally, the combination of HA-Gd_2_O_3_ NPs and X-ray irradiation had a synergistic effect on the cell colony formation, the cell viability in a dose-dependent manner, and radiosensitization enhancement effect. Based on these results, HA-Gd_2_O_3_ NPs may be an efficient alternative for enhancing radiosensitization for radiotherapy.

## Conclusions

In conclusion, we developed a bifunctional HA-Gd_2_O_3_ NPs for effective MR imaging and radiosensitization of tumors in a one-pot hydrothermal process. After coating with HA, the as-synthesized HA-Gd_2_O_3_ NPs exhibited favorable dispersibility in water, excellent biocompatibility. The resultant HA-Gd_2_O_3_ NPs encapsulating the Gd atoms not only effectively exhibited high longitudinal relaxivity (*r*_*1*_) for MRI as a *T*_1_ contrast agent, but also enhanced radiosensitivity of tumors by inducing cell apoptosis and blocking cell cycle as a radiosensitizer. Thus, the novel HA-Gd_2_O_3_ NPs show a promising potential for tumor diagnosis and radiotherapy.

## Materials and Methods

### Materials

Fluorescein diacetate (FDA) and propidium iodide (PI) were acquired from Sigma (New York, NY, USA). GdCl_3_·6H_2_O, ethylene glycol (99%) were purchased from the Hengrui Pharmaceutical Co., Ltd. (Lianyungang, Jiangsu, People’s Republic of China). The Cell Counting Kit-8 (CCK-8) was bought from Dojindo (Kumamoto, Japan). NaH_2_PO_4_, Na_2_HPO_4_, and H_2_SO_4_ were obtained from the Guangfu Fine Chemical Research Institute (Nankai, Tianjin, People’s Republic of China). Fetal bovine serum and Dulbecco’s minimum essential medium (DMEM) were purchased from the Invitrogen China Limited (Shanghai, People’s Republic of China). All chemicals were of analytical grade and were utilized without further purification.

### Synthesis of HA-Gd_2_O_3_ NPs

The HA-Gd_2_O_3_ NPs were prepared using one-pot hydrothermal approach as following: First, 0.1 g of HA was dissolved in 20 mL of water under vigorous stirring at ambient temperature overnight. Subsequently, 0.1 g of GdCl_3_·6H_2_O and 0.5 mL of NaOH (1 M) were added, respectively. Then, the mixture was agitated for another 5 min to form a homogeneous clear solution and transferred to a 50-mL autoclave, sealed, and hydrothermally treated at 120 °C for 6 h. After being cooled to room temperature naturally, the transparent suspension was filtered with a 0.22-μm membrane to remove any large-sized agglomeration. The prepared solution was then dialyzed against water for 3 days in a dialysis bag with 14-kDa molecular weight cutoff. The dialysis solution was collected and freeze-dried using a vacuum freeze dryer. The HA-Gd_2_O_3_ NP powders thus were obtained and saved for further characterization.

### Instrumentation and Characterizations

The morphologies of the HA-Gd_2_O_3_ NPs were examined by high-resolution transmission electron microscopy on a JEM-2100 microscope (JEOL, Tokyo, Japan) under an accelerating voltage of 200 kV. The elemental composition of HA-Gd_2_O_3_ NPs was determined by XPS measurements in a MK II photoelectron spectrometer, using Al-Ka (1486.6 eV) as the X-ray source and Fourier transform infrared (FTIR) spectrometer (Nicolet Nexus 470, GMI, Ramsey, MN, USA). The crystal structure of the HA-Gd_2_O_3_ NPs was characterized via X-ray diffraction (XRD) patterns on a Rigaku-D/MAX2500 diffractometer (Rigaku, Japan) equipped with Cu Kα (*λ* = 0.15405 nm) radiation at a scanning speed of 4°/min in the range from 5 to 80°.

### Cell Viability Assay

The influence of HA-Gd_2_O_3_ NPs on cell viability was studied via Cell Counting Kit CCK-8 assay (CCK-8 assay). HepG2 (human hepatocarcinoma, ATCC Number: HB-8065) and VSMCs (vascular smooth muscle cells) were seeded in a 96-well plate at a density of 3 × 10^3^ cells/well and cultured at 37 °C in 5% CO_2_ incubator for 24 h, then using DMEM containing different concentrations of HA-Gd_2_O_3_ NPs (0 μg/mL, 25 μg/mL, 50 μg/mL, 100 μg/mL, and 200 μg/mL) replacing the growth medium. After incubation for another 4 h, adding 10-μL CCK-8 solution to each well, cells were incubated for 4 h in a dark place. The absorbance was measured at 490 nm using Synergy HT Multi-Mode Microplate Reader (Bio Tek, Winooski, VT, USA). Non-treated cells (in DMEM) were used as the control, and the relative cell viability (mean SD, *n* = 5) was expressed as Abssample/Abscontrol × 100%.

### Hemolysis Assay

Briefly, 19–21 g, ages of 6-week female BALB/c mice were kindly prepared by the Animal Management Rules of the Ministry of Health of the People’s Republic of China. Three milliliters of the blood which stabilized with heparin sodium was obtained from removing eyeballs. Then, it was centrifuged to remove the supernatant with 1200 rpm, 15 min according to the literature. After that, washing the sedimentation with PBS for five times to obtain the mouse red blood cells (MRBCs), then taking about a 3-mL blood by removing the eyeball, stabilized with heparin sodium, centrifuged (1, 200 rpm, 15 min) to remove the supernatant according to the literature [27], washed with PBS for five times to obtain the mouse red blood cells (MRBCs). Diluting ten times with PBS, 0.1 mL MRBCs were transferred into 1.5 mL tubes prefilled with 0.9 mL PBS containing different particle concentrations (50–200 μg/mL) of HA-Gd_2_O_3_ NPs, 0.9 mL water (as positive control), and 0.9 mL PBS (as negative control), respectively. The mixture was incubated for 2 h at room temperature after a gentle shaking, then, centrifuged at 12,000 rpm for 1 min. Finally, the photographs of all the samples were taken and the absorbance of the supernatant (hemoglobin) was measured by a UV-2450 UV–Vis spectrophotometer. The hemolysis percentages of different samples were calculated by dividing the difference in absorbance among the sample, the positive, and negative control at 541 nm.

### MR Phantom Study In Vitro and In Vivo

In vitro and in vivo MR images were acquired on 3 Tesla MRI scanner (Magnetom Trio Tim, Siemens, Germany). To study the *T*_1_ phantom images in vitro, the solution of HA-Gd_2_O_3_ NPs with different concentrations ranging from 0 to 16 mM was added to a 96-well culture plate, using the Magnevist (commercial MR contrast agent, Gd-DTPA) as control. For MR imaging in vivo, we chose the normal BALB/c mice as the model (*n* = 4). Animal experiments were strictly conformed to the Animal Management Rules of the Ministry of Health of the People’s Republic of China. Ten milligrams per kilogram of HA-Gd_2_O_3_ NPs filtering through sterilized membrane filters (pore size 0.22 μm) were intravenously injected into the animals, then immediately investigated with a MRI scanner. These samples for MR imaging in vitro and animals in vivo were imaged with the following parameters: TR/TE = 300/10 ms, 256 × 256 matrices, slices = 5, thickness = 2 mm, averages = 2, FOV = 80 × 80.

### Radiosensitization Effect In Vitro

CCK-8 assay was used to evaluate radiosensitizing activity of HA-Gd_2_O_3_ NPs in vitro. Cells were seeded in five 96-well plates at a density of 3 × 10^3^ cells/well, and each plate was treated at the same condition: cultured at 37 °C in 5% CO_2_ incubator for 24 h, then using DMEM containing different concentrations of HA-Gd_2_O_3_ NPs (0 μg/mL, 12.5 μg/mL, 25 μg/mL, 50 μg/mL, 100 μg/mL, and 200 μg/mL) replacing the growth medium. After incubation for 4 h, five plates were irradiated at different X-ray doses (0 Gy, 3 Gy, 6 Gy, and 9 Gy), 300 cGy/min, respectively. After radiation, all the plates were incubated for 4 h, then measuring the absorbance under the same parameters.

Clonogenic survival assay was also conducted to study the radiosensitization effect in vitro. HepG2 cells were seeded in six-well plates at 4.0 × 10^4^ cells/well and allowed to grow for 16 h. The cells were incubated with HA-Gd_2_O_3_ NPs diluted in cell culture medium for 6 h. The cells were then irradiated at 6 Gy using a clinical linear accelerator (Oncor, Siemens, Germany) with 6 MeV irradiation using a 10 cm × 10 cm radiation field at a source-to-skin distance (SSD) of 100 cm to cover the entire cells. Then, these irradiated cells were allowed to grow for 14 days, fixed with 4% paraformaldehyde at room temperature for 40 min, stained with a 1% crystal violet after washing the cells.

### Live-Dead Staining Assay and Flow Cytometry

To study the radiosensitization effect of HA-Gd_2_O_3_ NPs, HepG2 cells were seeded in six-well plates at a density of 4.0 × 10^4^ cells/well and allowed to grow for 12 h and set six groups (control, HA-Gd_2_O_3_ NPs, radiation, radiation + 50 μg/mL HA-Gd_2_O_3_ NPs, radiation + 100 μg/mL HA-Gd_2_O_3_ NPs and radiation + 200 μg/mL HA-Gd_2_O_3_ NPs). When cells were grown to 80% in plates, the first group had no treatment, the second group was incubated with 100 μg/mL HA-Gd_2_O_3_ NPs for 24 h, the third group was just irradiated, and the fourth group to the sixth group were irradiated and incubated with different concentrations of HA-Gd_2_O_3_ NPs (50, 100, and 200 μg/mL) for 24 h, respectively. After that, FDA and PI working buffer were added for cell staining. The fluorescence of stained cells was observed under a fluorescence microscope (×20), and live cells showed green color and dead ones exhibited red color. Furthermore, cells by different treatments were washed three times with PBS, digested, collected, and centrifuged at a speed of 2000 rpm for 5 min, then fixed with 70% ethanol at − 20 °C overnight followed by PI staining. DNA fragmentation was quantified by the fluorescence intensity of PI on a flow cytometer (BD, Accuri, C6BD, Accuri, C6), analyzed by software (FLOWJO 7. 6. 2) to make clear the cell cycle distribution.

### Radiosensitization Effect In Vivo

Female BALB/c mice with body weights of 19–21 g and ages of 6 weeks were obtained from the Yangzhou University Laboratory Animal Center under the standard conditions. Animal experiments were compliant with the Animal Management Rules of the Ministry of Health of the People’s Republic of China. A subcutaneous tumor model was established as the following procedures: First, 1 × 10 ^6^ HepS cells were inoculated into mice intraperitoneal, and the ascites were collected after 5 days. Then, these ascites were injected into subcutaneous. When the tumor sizes reached approximately 100 mm^3^, subcutaneous tumors models were established and applied to the following experiments.

Eight mice bearing subcutaneous tumors per group were treated with radiation at 3 Gy per fraction to a total dose of 9 Gy within 7 days. The radiotherapy was conducted after 3 h intravenous injection of HA-Gd_2_O_3_ NPs (10 mg/Kg), on a Siemens Primus clinical linear accelerator (6 MeV) using a self-made device to cover the entire tumor. These mice were anesthetized by 1% pentobarbital (50 mg/kg) to assure immobility during irradiation. The volume was measured and recorded every day, determined from caliper measurement, and calculated by the formulae: *V* = 0.5 × *a* × *b*^2^, where *V* (mm^3^) is the volume of the tumor, and *a* (mm) and *b* (mm) are the tumor length and tumor width, respectively. Relative tumor volumes were normalized to their initial sizes. Each group was conducted on eight mice, wherein statistical analysis was performed using Student’s two-tailed *t* test (*p < 0.05, **p < 0.001).

## Supplementary information

**Additional file 1: Figure S1.** The characterization of biocompatibility of HA-Gd_2_O_3_ NPs using CCK-8 assay.

**Additional file 2: Figure S2.** H&E stained histological images of spleen, kidney, and bladder after intravanous injection of HA-Gd2O3 NPs. Bar: 100 μm.

## Data Availability

The data sets supporting the results of this article are included within the article.
