# Supplementary figures and images for: Hyaluronic Acid-Functionalized Gadolinium Oxide Nanoparticles for Magnetic Resonance Imaging-Guided Radiotherapy of Tumors
Source: Nanoscale Res Lett. 2020 Apr 25;15:94. doi: 10.1186/s11671-020-03318-9 (PMC7183523; doi:10.1186/s11671-020-03318-9)

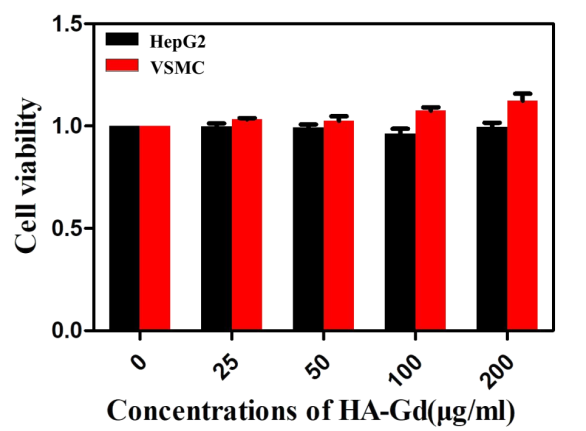

Supplement: Supplementary file 1 — Additional file 1: Figure S1. The characterization of biocompatibility of HA-Gd2O3 NPs using CCK-8 assay. [file 11671_2020_3318_MOESM1_ESM.docx]

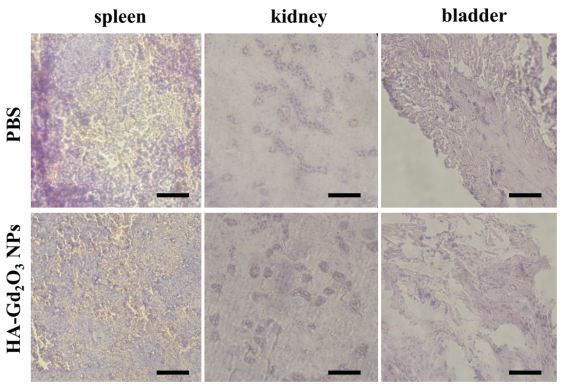

Supplement: Supplementary file 2 — Additional file 2: Figure S2. H&E stained histological images of spleen, kidney, and bladder after intravanous injection of HA-Gd2O3 NPs. Bar: 100 μm. [file 11671_2020_3318_MOESM2_ESM.docx]
